# Quality of Life and Awareness of Hypertension Among Hypertensive Patients in Saudi Arabia

**DOI:** 10.7759/cureus.14879

**Published:** 2021-05-06

**Authors:** Sulaiman A Alshammari, Anwar N Alajmi, Rema A Albarrak, Alaa B Alaqil, Ghaida K Alsaeed, Muneerah Z Alzayed, Hamdan N Alajami, Jaffer B Baqar, Sheraz Ali

**Affiliations:** 1 Family and Community Medicine, King Saud University, Riyadh, SAU; 2 College of Medicine, King Saud University, Riyadh, SAU; 3 Pharmaceutical Care Services, King Saud Medical City, Riyadh, SAU; 4 Statistics, University of Karachi, Karachi, PAK; 5 School of Pharmacy and Pharmacology, University of Tasmania, Hobart, AUS

**Keywords:** hypertension in the global context, heath related quality of life, patient knowledge, clinic blood pressure, keywords awareness

## Abstract

Background and aim

Hypertension possesses significant public health challenges for both developing and developed economies. Therefore, it is crucial to evaluate the awareness of hypertension and health-related quality of life (HRQoL) among patients with hypertension. This study aims to evaluate the knowledge of hypertension and HRQoL among hypertensive patients.

Materials and methods

A cross-sectional study using an anonymous questionnaire was conducted over a period of 4 months (November 2020 to February 2021) in Riyadh, Kingdom of Saudi Arabia (KSA). This study included 437 questionnaires submitted by the hypertensive population.

Results

A total of 437 participants were included in this study, with 55.4% being males (n=242). The majority (85.1%) were aware of the normal values of blood pressure. Many participants had no problems with mobility, personal care, usual activities, pain or discomfort, and anxiety or depression. Age groups showed a significant association with mobility and usual activities. The knowledge of hypertension was significantly associated with HRQoL.

Conclusion

This study found that majority of the hypertensive patients were aware of the standard values of blood pressure. HRQoL concerning physical functioning and general health is found to be good based on the EuroQol visual analogue scale (EQ VAS) in the hypertensive population who are aware of their condition. This study reported a weak yet significant association between hypertension-related knowledge and HRQoL scores. Several factors can affect the HRQoL of the hypertensive population including gender, education, occupation, and income status.

## Introduction

Hypertension (defined as systolic blood pressure above 140 mmHg with a diastolic blood pressure of less than 90 mmHg) is quantitatively the most important modifiable risk factor of cardiovascular and kidney disease, and a leading risk factor for mortality [1]. It causes significant public health challenge for both developing and developed economies [2]. According to the World Health Organization, an estimated 1.13 billion people worldwide have hypertension [3]. Globally, seven million people die each year because of hypertension [4]. The prevalence of hypertension in the Kingdom of Saudi Arabia (KSA) is 25.5% among those aged 15-64 years [5-7]. The most recent study revealed that the prevalence of hypertension among the Saudi population is 31.4% [8]. A nationwide survey in the KSA revealed that 57.8% of those with hypertension were not aware of their condition [9]. Lack of information and poor self-care practice increases the burden of hypertension and are a major challenge in controlling hypertension [10]. In the KSA, hypertension is an important public health problem and has become a leading risk factor for death [11].

Health-related quality of life (HRQoL) is defined as “an individual’s or a group’s perceived physical and mental health over time” [12]. HRQoL is a multi-dimensional concept and critical indicator of an individual’s overall health as it captures information on the physical and mental health state of individuals and on the impact of health status on quality of life [13,14]. Hypertension adversely affects a patient’s quality of life [15]. Previous studies have also shown a significant reduction in the HRQoL in hypertensive population to be associated with patient health comorbidities, knowledge of diagnosis, and adverse effects from medications [16].

Given the high prevalence of hypertension and lack of awareness, the impact on patient’s quality of life is inevitable. There is a growing demand to increase awareness among patients about chronic diseases [17]. Several strategies have earlier been implemented to improve patient’s knowledge including patient groups, published scientific literature, specialists’ clinics, and the uptake of information technology [18]. The provision of disease-related knowledge to the population is regarded as a good practice; however, the impact of this knowledge on HRQoL is uncertain. Furthermore, no specific information about the impact of hypertension-related awareness on HRQoL for the hypertensive Saudi population exists. Therefore, this study aims to evaluate the knowledge of hypertension and HRQoL among hypertensive patients. In addition, we also examined the association between HRQoL and hypertension-related knowledge among the hypertensive population in the KSA. The study will help determine the factors compromising the quality of the life and facilitate the designing and implementation of customized educational programs for the hypertensive population, thereby improving the HRQoL.

## Materials and methods

This cross-sectional study was conducted among hypertensive Saudi patients from November 2020 to February 2021 at the outpatient clinic of the King Saud University Medical City, a tertiary care setting in Riyadh, KSA. This medical city serves a broad range of patients drawn from a large population, several of whom present with complex medical comorbidities.

Patients aged 18 years and over, with a confirmed diagnosis (blood pressure reading was taken on two separate occasions that is greater than 140/90 mmHg) of essential hypertension, who have been using antihypertensive agents for the previous three months were included in the study. Patients aged < 18 and > 80 years, having co-morbidities, expatriates from other countries, and pregnant women were excluded.

A 21-item self-administered questionnaire for assessing the knowledge of hypertension was distributed among hypertensive patients at the King Saud University Medical City. This questionnaire was adopted from previous studies [19]. The questionnaire was used to collect demographics, duration of disease and assess hypertensive patient’s knowledge toward causes, treatment, and management of hypertension. The assessment of the knowledge was primarily based on the knowledge about normal values of blood pressure.

Data for the quality of life was collected using EuroQol five-dimensions - 3-level (EQ-5D-3L), the European quality of life scale [20]. EQ-5D-3L consists of two sections: the EQ-5D descriptive system and the EQ visual analogue scale (EQ VAS). The EQ-5D-3L descriptive system comprises the following five dimensions: mobility, self-care, usual activities, pain or discomfort, and anxiety or depression. Each dimension has the following three levels: no problems, some or moderate problems, and extreme problems. The hypertensive patient was asked to indicate his or her health state by selecting the box next to the most appropriate statement in each of the five dimensions. The EQ VAS records the patient’s self-rated health on a 20 cm vertical visual analogue scale with two distinct endpoints such as "Best imaginable health state" for a score of 100 and "Worst imaginable health state" for a score of 0. The VAS is used as a quantitative measure of health outcome that reflects the subject’s own judgement. All instruments were pre-tested for reliability and validity. The translated Arabic version of EQ-5D-3L was provided by the Euroqol after registering this study on Euroqol’s website. All participants were briefed about the purpose of the study and were also provided with a study information sheet; Informed consent was obtained from all the study participants. This study was initiated after the approval of the Institutional Review Board of King Saud University Medical City (Reference number: E-20-5457).

In order to elicit a response in 50% of respondents regarding the knowledge of hypertension, with a 95% confidence level and 5% margin of error, a sample size of 377 was estimated. The sample size was calculated by an online sample size calculator. Data analysis was carried out using the SPSS software, version 24 (IBM. Armonk, NY: IBM Corp). Numerical data were presented with mean and standard deviation whereas categorical data in frequencies and percentages. Independent samples’ t-test and analysis of variance (ANOVA) were applied for two and more than two means difference, respectively. EQ-5D-3L scores for five dimensions and mean EQ VAS scores were also compared for age and gender. Pearson correlation was used to explore any correlations between knowledge and HRQoL. Correlations were interpreted using the following criteria: 0 to 0.25 = weak correlation, 0.25 to 0.5 = fair correlation, 0.5 to 0.75 = good correlation and > 0.75 = excellent correlation [21]. A P-value of <0.05 was taken as the level of significance between responses.

## Results

A total of 437 questionnaires were returned by the hypertensive patients, and the response rate was 87.4%. The majority of the patients belong to the age group of 48 years and over, with 55.4% being males (n=242). Nearly 47% of the study participants had a bachelor’s degree. Twenty-six percent (n=115) had a monthly income of more than 15,000 Saudi riyals. Ninety-seven percent of the study participants resided in urban settings and 3% were from rural areas. Nearly 36% had a duration of hypertension for more than 15 years. There was a significant association in EQ-VAS scores with respect to gender, qualification, occupation, monthly income, and the duration of disease. The mean EQ-VAS score (77.1 ± 17.6) indicated good HRQoL in our study participants (Table 1).

**Table 1:** Mean EQ VAS scores among study demographics. EQ VAS: EuroQol visual analogue scale.

Variables	n (n%)	EQ VAS score (mean ±SD)	P-value
Age groups (years)			
18-27	03 (0.7)	80 ± 10	0.203
28-37	12 (2.7)	81.7 ± 11.9	
38-47	57 (13)	81.1 ± 18.1	
48 & above	365 (83.5)	76.3 ± 17.7	
Gender			
Male	242 (55.4)	80.5 ± 15.3	<0.001
Female	195 (44.6)	72.7 ± 19.3	
Education			
No formal education	37 (8.5)	62.2 ± 25.4	<0.001
Primary	58 (13.3)	76.2 ± 17.4	
Intermediate	84 (19.2)	74.8 ± 17.2	
Bachelors	203 (46.5)	79.6 ± 15.6	
Masters	55 (12.6)	82 ± 13.5	
Occupation			
Retired	146 (33.4)	77.7 ± 16.1	<0.001
Jobless	146 (33.4)	72.5 ± 20.1	
Government job	136 (31.1)	81.3 ± 15.4	
Businessman	09 (2.1)	77.8 ± 15.6	
Income			
Nill	91 (20.8)	72.8 ± 19.6	<0.001
Less than 5,000	59 (13.5)	68.9 ± 20.2	
5,000-10,000	88 (20.1)	79.7 ± 16.8	
10,001-15,000	84 (19.2)	80.7 ± 13.8	
Above 15,000	115 (26.3)	80 ± 15.6	
Locality			
Urban	424 (97)	77.3 ± 17.4	0.104
Rural	13 (3)	69.2 ± 22.2	
Duration of disease (years)			
<1	15 (3.4)	75.3 ± 22.0	0.024
1-3	42 (9.6)	81.4 ± 14.6	
4-5	61 (14)	82.3 ± 16.4	
6-15	162 (37.1)	76.4 ± 16.8	
>15	157 (35.9)	74.7 ± 18.7	
Overall EQ VAS score (mean ± SD): 77.1 ± 17.6			

More than half of the participants (55.4%) were not aware of the basic definition of hypertension; however, the majority (85.1%) were aware of the normal values of blood pressure (Table 2). More than 80% correctly identified smoking, fatty food, and obesity as risk factors of hypertension. Many study participants (93.4%) were aware that hypertension can lead to other life-threatening diseases.

**Table 2:** Comparison of responses about hypertension knowledge among age groups and gender.

Variables	Overall	Age groups (years)			Gender		
		18-47	48 & above	P-value	Male	Female	P-value
	(n=437)	(n=72)	(n=365)		(n=242)	(n=195)	
Q1: Know the normal values of blood pressure							
Yes	372 (85.1)	62 (86.1)	310 (84.9)	0.797	222 (91.7)	150 (76.9)	<0.001
No	65 (14.9)	10 (13.9)	55 (15.1)		20 (8.3)	45 (23.1)	
Q2: Elevated blood pressure is called hypertension							
Yes	167 (38.2)	30 (41.7)	137 (37.5)	<0.001	109 (45)	58 (29.7)	0.001
No	28 (6.4)	12 (16.7)	16 (4.4)		10 (4.1)	18 (9.2)	
Don’t know	242 (55.4)	30 (41.7)	212 (58.1)		123 (50.8)	119 (61)	
Q3: Hypertension can progress with age							
Yes	213 (48.7)	33 (45.8)	180 (49.3)	0.857	126 (52.1)	87 (44.6)	0.251
No	36 (8.2)	6 (8.3)	30 (8.2)		17 (7)	19 (9.7)	
Don’t know	188 (43)	33 (45.8)	155 (42.5)		99 (40.9)	89 (45.6)	
Q4: Both genders have equal chance of developing hypertension							
Yes	107 (24.5)	22 (30.6)	85 (23.3)	0.423	52 (21.5)	55 (28.2)	0.072
No	113 (25.9)	17 (23.6)	96 (26.3)		72 (29.8)	41 (21)	
Don’t know	217 (49.7)	33 (45.8)	184 (50.4)		118 (48.8)	99 (50.8)	
Q5: HTN is a treatable condition							
Yes	258 (59)	38 (52.8)	220 (60.3)	0.268	144 (59.5)	114 (58.5)	0.46
No	60 (13.7)	14 (19.4)	46 (12.6)		29 (12)	31 (15.9)	
Don’t know	119 (27.2)	20 (27.8)	99 (27.1)		69 (28.5)	50 (25.6)	
Q6: The older a person is, greater risk of having hypertension							
Yes	345 (78.9)	56 (77.8)	289 (79.2)	0.097	188 (77.7)	157 (80.5)	0.768
No	31 (7.1)	9 (12.5)	22 (6)		18 (7.4)	13 (6.7)	
Don’t know	61 (14)	7 (9.7)	54 (14.8)		36 (14.9)	25 (12.8)	
Q7: Smoking is a risk factor for hypertension							
Yes	357 (81.7)	68 (94.4)	289 (79.2)	0.008	199 (82.2)	158 (81)	0.867
No	10 (2.3)	0	10 (2.7)		6 (2.5)	4 (2.1)	
Don’t know	70 (16)	4 (5.6)	66 (18.1)		37 (15.3)	33 (16.9)	
Q8: Eating fatty food is a risk factor for hypertension							
Yes	406 (92.9)	72 (100)	334 (91.5)	0.037	229 (94.6)	177 (90.8)	0.199
No	05 (1.1)	0	5 (1.4)		3 (1.2)	2 (1)	
Don’t know	26 (5.9)	0	26 (7.1)		10 (4.1)	16 (8.2)	
Q9: Overweight increases risk for hypertension							
Yes	406 (92.9)	72 (100)	334 (91.5)	0.037	226 (93.4)	180 (92.3)	0.200
No	06 (1.4)	0	6 (1.6)		5 (2.1)	1 (0.5)	
Don’t know	25 (5.7)	0	25 (6.9)		11 (4.5)	14 (7.2)	
Q10: Regular physical activity lowers a chance of hypertension							
Yes	384 (87.9)	68 (94.4)	316 (86.6)	0.142	223 (92.1)	161 (82.6)	0.006
No	16 (3.7)	2 (2.8)	14 (3.8)		4 (1.7)	12 (6.2)	
Don’t know	37 (8.5)	2 (2.8)	35 (9.6)		15 (6.2)	22 (11.3)	
Q11: Eating more salt has no effect on blood pressure							
Yes	30 (6.9)	4 (5.6)	26 (7.1)	0.863	18 (7.4)	12 (6.2)	0.274
No	374 (85.6)	63 (87.5)	311 (85.2)		210 (86.8)	164 (84.1)	
Don’t know	33 (7.6)	5 (6.9)	28 (7.7)		14 (5.8)	19 (9.7)	
Q12: Dietary approaches to reduce hypertension do no good							
Yes	82 (18.8)	16 (22.2)	66 (18.1)	0.129	37 (15.3)	45 (23.1)	0.116
No	300 (68.6)	52 (72.2)	248 (67.9)		173 (71.5)	127 (65.1)	
Don’t know	55 (12.6)	4 (5.6)	51 (14)		32 (13.2)	23 (11.8)	
Q13: White meat is as good as red meat in hypertension							
Yes	130 (29.7)	24 (33.3)	106 (29)	0.686	73 (30.2)	57 (29.2)	0.005
No	176 (40.3)	26 (36.1)	150 (41.1)		111 (45.9)	65 (33.3)	
Don’t know	131 (30)	22 (30.6)	109 (29.9)		58 (24)	73 (37.4)	
Q14: Medication alone can control hypertension							
Yes	53 (12.1)	5 (6.9)	48 (13.2)	0.123	32 (13.2)	21 (10.8)	0.624
No	350 (80.1)	64 (88.9)	286 (78.4)		193 (79.8)	157 (80.5)	
Don’t know	34 (7.8)	3 (4.2)	31 (8.5)		17 (7)	17 (8.7)	
Q15: Hypertension can lead to other life-threatening diseases							
Yes	408 (93.4)	70 (97.2)	338 (92.6)	0.342	230 (95)	178 (91.3)	0.139
No	02 (0.5)	0	2 (0.5)		0	2 (1)	
Don’t know	27 (6.2)	2 (2.8)	25 (6.9)		12 (5)	15 (7.7)	

Table 3 and Table 4 report HRQoL scores among study participants. Regarding the five dimensions of EQ-5D-3L, many participants had no problems with mobility (63.6%), personal care (93.4%), usual activities (74.4%), pain or discomfort (54.5%), and anxiety or depression (65.7%); however, age groups showed a significant association with mobility and usual activities. Both mobility and usual activities had a significant association with age and gender. In contrast, personal care (P < 0.001), pain or discomfort (P < 0.001), and anxiety or depression (P = 0.009) showed a significant association with gender (Table 3).

**Table 3:** Association of five dimensions of EQ-5D-3L with age groups and gender. EQ-5D-3L: EuroQol five-dimensions-three-level.

Variables	Overall	Age groups (years)			Gender		
		18-47	48 & above	P-value	Male	Female	P-value
	(n=437)	(n=72)	(n=365)		(n=242)	(n=195)	
Mobility							
I have no problems with walking around	278 (63.6)	62 (86.1)	216 (59.2)	<0.001	184 (76)	94 (48.2)	<0.001
I have some problems with walking around	154 (35.2)	8 (11.1)	146 (40)		54 (22.3)	100 (51.3)	
I am confined to bed	5 (1.1)	2 (2.8)	3 (0.8)		4 (1.7)	1 (0.5)	
Personal care							
I have no problems with washing or dressing myself	408 (93.4)	66 (91.7)	342 (93.7)	0.384	233 (96.3)	175 (89.7)	0.015
I have some problems with washing or dressing myself	20 (4.6)	3 (4.2)	17 (4.7)		5 (2.1)	15 (7.7)	
I am unable to wash or dress myself	9 (2.1)	3 (4.2)	6 (1.6)		4 (1.7)	5 (2.6)	
Usual activities (e.g., work, study, housework, family or leisure activities)							
I have no problems with performing my usual activities	325 (74.4)	65 (90.3)	260 (71.2)	0.003	213 (88)	112 (57.4)	<0.001
I have some problems with performing my usual activities	95 (21.7)	6 (8.3)	89 (24.4)		22 (9.1)	73 (37.4)	
I am unable to perform my usual activities	17 (3.9)	1 (1.4)	16 (4.4)		7 (2.9)	10 (5.1)	
Pain/discomfort							
I have no pain or discomfort	238 (54.5)	46 (63.9)	192 (52.6)	0.184	172 (71.1)	66 (33.8)	<0.001
I have moderate pain or discomfort	186 (42.6)	25 (34.7)	161 (44.1)		67 (27.7)	119 (61)	
I have extreme pain or discomfort	13 (3)	1 (1.4)	12 (3.3)		3 (1.2)	10 (5.1)	
Anxiety/depression							
I am not anxious or depressed	287 (65.7)	46 (63.9)	241 (66)	0.785	174 (71.9)	113 (57.9)	0.009
I am moderately anxious or depressed	142 (32.5)	24 (33.3)	118 (32.3)		64 (26.4)	78 (40)	
I am extremely anxious or depressed	8 (1.8)	2 (2.8)	6 (1.6)		4 (1.7)	4 (2.1)	

**Table 4:** Mean EQ VAS score comparison and correlation of EQ-5D levels with responses about hypertension knowledge. EQ VAS: EuroQol visual analogue scale; EQ-5D: EuroQol five-dimensions.

Variables	EQ VAS score		Mobility		Personal care		Usual activities		Pain/discomfort		Anxiety/depression	
	Mean ±SD	P-value	r	P-value	r	P-value	r	P-value	r	P-value	r	P-value
Know the normal values of blood pressure												
Yes	78.4 ± 16.6	<0.001	0.17	<0.001	0.2	<0.001	0.2	<0.001	0.2	<0.001	0.14	0.004
No	69.3 ± 21.0											
Elevated blood pressure is called hypertension												
Yes	76.8 ± 18.1	0.028	0.02	0.672	0.02	0.615	0.04	0.4	0.08	0.085	0.04	0.466
No	84.6 ± 12.3											
Hypertension can progress with age												
Yes	77.3 ± 16.8	0.736	0.03	0.486	0.01	0.885	0.03	0.491	0.04	0.434	-0.07	0.137
No	78.4 ± 15.4											
Both genders have equal chance of developing hypertension												
Yes	78.2 ± 17.4	0.564	0.06	0.196	0.07	0.146	0.05	0.325	0.07	0.122	-0.01	0.88
No	76.9 ± 16.5											
Hypertension is a treatable condition												
Yes	77.5 ± 17.9	0.552	-0.03	0.545	-0.02	0.632	0.03	0.491	0.03	0.483	-0.03	0.578
No	76.0 ± 17.8											
The older a person is, the greater risk of having hypertension												
Yes	77.2 ± 17.6	0.509	0.04	0.411	-0.01	0.938	0.05	0.342	0.02	0.678	-0.04	0.36
No	79.4 ± 11.8											
Smoking is a risk factor for hypertension												
Yes	78.3 ± 16.9	0.058	0.21	<0.001	-0.01	0.861	0.11	0.024	0.14	0.003	-0.01	0.793
No	68.0 ± 20.4											
Eating fatty food is a risk factor for hypertension												
Yes	77.2 ± 17.3	0.54	0.09	0.073	0.04	0.464	0.04	0.361	0.05	0.297	-0.09	0.064
No	82.0 ± 14.8											
Overweight increases risk for hypertension												
Yes	77.6 ± 17.2	0.123	0.07	0.164	-0.07	0.124	0.08	0.095	0.06	0.22	0.04	0.36
No	66.7 ± 15.1											
Regular physical activity lowers a chance of hypertension												
Yes	77.4 ± 17.3	0.9	0.09	0.076	-0.02	0.73	0.1	0.036	0.08	0.089	0.019	0.685
No	76.9 ± 17.4											
Eating more salt has no effect on blood pressure												
Yes	79.7 ± 17.5	0.418	0.06	0.207	0.02	0.698	0.09	0.075	0.13	0.008	0.02	0.739
No	77.1 ± 17.1											
Dietary approaches to reduce hypertension do no good												
Yes	74.9 ± 17.4	0.17	0.01	0.816	-0.07	0.131	-0.02	0.757	-0.04	0.4	-0.08	0.097
No	77.8 ± 17.1											
White meat is as good as red meat in hypertension												
Yes	77.5 ± 18.4	0.631	0.05	0.26	0.03	0.497	0.03	0.539	0.09	0.057	-0.03	0.607
No	78.4 ± 15.9											
Medication alone can control hypertension												
Yes	80.2 ± 15.4	0.231	0.07	0.175	0.01	0.937	0.07	0.127	0.04	0.383	0.0	0.999
No	77.2 ± 17.1											
Hypertension can lead to other life-threatening diseases												
Yes	77.3 ± 17.1	0.523	-0.01	0.919	-0.03	0.501	0.11	0.023	0.07	0.152	-0.06	0.22
No	85.0 ± 7.1											

**Figure 1:**
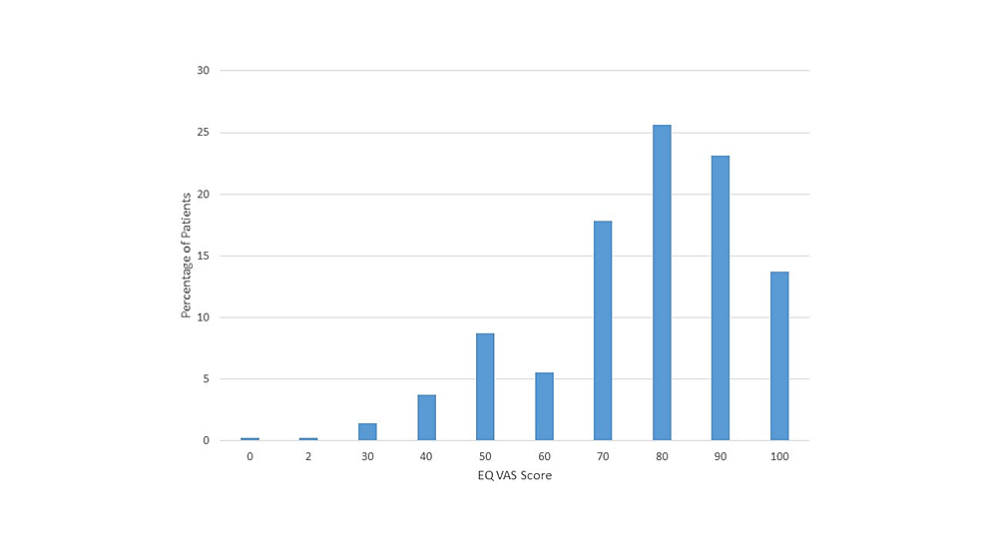
EQ VAS health index of study participants. EQ VAS: EuroQol visual analogue scale.

The score of HRQoL based on EQ-VAS score range from 0 (worst imaginable health state) to 100 (best imaginable health state) (Figure 1).

Table 4 showed the correlation between HRQoL and knowledge of hypertension. The study findings revealed that the main variables of the knowledge about hypertension such as normal values (P <0.001) and the definition (P = 0.028) of hypertension were significantly associated with HRQoL (mean VAS score) (Table 4). Pearson correlation test showed that the knowledge about normal values of blood pressure and HRQoL scores had a weak correlation. The knowledge about the definition of hypertension and HRQoL scores also showed a weak correlation.

## Discussion

To our knowledge, this is the first study conducted in the KSA using EQ-5D-3L to measure HRQoL among hypertensive patients visiting outpatient clinics. Moreover, the relationship between the knowledge of hypertension and HRQoL has not been explored among the Saudi population. This study reports that many hypertensive patients, particularly those living in urban areas, had knowledge about the standard values of blood pressure. Similarly, the findings of this study highlight that the knowledge of hypertension (based on the normal values of blood pressure) is significantly associated with HRQoL, although a weak correlation. This finding is consistent with that of other studies conducted in hypertensive population in Pakistan [22]; however, our study is relatively larger in sample size, and also informs about individualized factors affecting HRQoL in hypertensive patients.

Previous studies provide inconsistent findings ranging from weak to strong correlation between the quality of life among hypertensive population and adherence to treatment [23,24]. The differences in the findings are probably due to the measurement of HRQoL using different instruments such as the World Health Organization Quality of Life Instrument (WHOQOL-BREF), EQ-5D-3L, Short Form 36 (SF36), or SF-12 [25]. Our results in the outpatient study population indicate that the HRQoL is good in the majority of hypertensive patients who are aware of the normal values of blood pressure. Education has been recognized as an important determinant of HRQoL; patients with higher educational levels usually report better HRQoL [26]. Nearly 60% of the hypertensive participants in our study had higher education. Previous studies reported better HRQoL among highly educated hypertensive patients [27]. Patients with higher education demonstrate good physical function through acquiring knowledge about appropriate health practices [28]. The results of this study also refute the assumption that disease-related awareness can decrease HRQoL particularly in patients suffering from chronic conditions such as hypertension and diabetes mellitus, due to their impact on psychosocial domains [29]. Likewise, the provision of education programs in previous studies demonstrated either a decline or no improvement in HRQoL scores [30]. Numerous methods have been utilized to improve patient knowledge such as specialist clinics, scientific literature, and information technology [18].

Strengths of our study include patients with a confirmed diagnosis of essential hypertension who have been using antihypertensive agents for the previous three months, the EQ-5D-3L questionnaire which is the European quality of life scale, and exclusion of complications of hypertension that might reduce HRQoL. In addition, we included a large sample of hypertensive patients, and determined individualized factors affecting HRQoL in the hypertensive population. There were some limitations to this study. First, this study was cross-sectional and limited in its capacity to inform about causality. It was conducted at a single tertiary care setting in the KSA, the results cannot be generalized to the entire population. Therefore, these results need to be validated by a multicentre longitudinal study across tertiary care settings in the Kingdom.

## Conclusions

This study found that majority of the hypertensive patients were aware of the standard values of blood pressure. HRQoL concerning physical functioning and general health found to be good based on EQ VAS in the hypertensive population who are aware of their condition. This study reported a weak yet significant association between hypertension-related knowledge and HRQoL scores. Several factors can affect the HRQoL of the hypertensive population including gender, education, occupation, and income status. The quality of life among hypertensive patients can be improved by conducting educational programs about hypertension at the community level. Future studies can explore the effect of an interdisciplinary education programs on hypertensive patient’s outcomes.
